# Aminoterminal Amphipathic α-Helix AH1 of Hepatitis C Virus Nonstructural Protein 4B Possesses a Dual Role in RNA Replication and Virus Production

**DOI:** 10.1371/journal.ppat.1004501

**Published:** 2014-11-13

**Authors:** Jérôme Gouttenoire, Roland Montserret, David Paul, Rosa Castillo, Simon Meister, Ralf Bartenschlager, François Penin, Darius Moradpour

**Affiliations:** 1 Division of Gastroenterology and Hepatology, Centre Hospitalier Universitaire Vaudois, University of Lausanne, Lausanne, Switzerland; 2 Institut de Biologie et Chimie des Protéines, Bases Moléculaires et Structurales des Systèmes Infectieux, UMR 5086, CNRS, Labex Ecofect, University of Lyon, Lyon, France; 3 Department of Infectious Diseases, Molecular Virology, University of Heidelberg, Heidelberg, Germany; The University of Chicago, United States of America

## Abstract

Nonstructural protein 4B (NS4B) is a key organizer of hepatitis C virus (HCV) replication complex formation. In concert with other nonstructural proteins, it induces a specific membrane rearrangement, designated as membranous web, which serves as a scaffold for the HCV replicase. The N-terminal part of NS4B comprises a predicted and a structurally resolved amphipathic α-helix, designated as AH1 and AH2, respectively. Here, we report a detailed structure-function analysis of NS4B AH1. Circular dichroism and nuclear magnetic resonance structural analyses revealed that AH1 folds into an amphipathic α-helix extending from NS4B amino acid 4 to 32, with positively charged residues flanking the helix. These residues are conserved among hepaciviruses. Mutagenesis and selection of pseudorevertants revealed an important role of these residues in RNA replication by affecting the biogenesis of double-membrane vesicles making up the membranous web. Moreover, alanine substitution of conserved acidic residues on the hydrophilic side of the helix reduced infectivity without significantly affecting RNA replication, indicating that AH1 is also involved in virus production. Selective membrane permeabilization and immunofluorescence microscopy analyses of a functional replicon harboring an epitope tag between NS4B AH1 and AH2 revealed a dual membrane topology of the N-terminal part of NS4B during HCV RNA replication. Luminal translocation was unaffected by the mutations introduced into AH1, but was abrogated by mutations introduced into AH2. In conclusion, our study reports the three-dimensional structure of AH1 from HCV NS4B, and highlights the importance of positively charged amino acid residues flanking this amphipathic α-helix in membranous web formation and RNA replication. In addition, we demonstrate that AH1 possesses a dual role in RNA replication and virus production, potentially governed by different topologies of the N-terminal part of NS4B.

## Author Summary

With an estimated 180 million chronically infected individuals, hepatitis C virus (HCV) is a leading cause of chronic hepatitis, liver cirrhosis and hepatocellular carcinoma worldwide. HCV is a positive-strand RNA virus that builds its replication complex on rearranged intracellular membranes, designated as membranous web. HCV nonstructural protein 4B (NS4B) is a key organizer of HCV membranous web and replication complex formation. Here, we provide a detailed structure-function analysis of an N-terminal amphipathic α-helix of NS4B, named AH1, and demonstrate that it plays key roles in shaping the membranous web as well as in virus production. We also show that the N-terminal part of NS4B adopts a dual membrane topology in a replicative context, possibly reflecting the different roles of this protein in the viral life cycle.

## Introduction

Hepatitis C virus (HCV) infection is a leading cause of chronic hepatitis, liver cirrhosis and hepatocellular carcinoma worldwide, with a peak of the disease burden expected in around 10 years from now [1]. HCV and GB virus B have been classified in the *Hepacivirus* genus within the *Flaviviridae* family, which also includes the genera *Flavivirus*, *Pestivirus* and *Pegivirus* [2]. Additional closely related viruses have been identified recently in horses as well as other animal species, and have been classified in the *Hepacivirus* and *Pegivirus* genera, including nonprimate hepaciviruses (NPHV) [3], [4].

HCV contains a 9.6-kb positive-strand RNA genome encoding a polyprotein precursor that is co- and posttranslationally processed into ten structural and nonstructural proteins [2], [5]. As all positive-strand RNA viruses, HCV replicates its genome in a membrane-associated replication complex composed of viral proteins, replicating RNA, rearranged intracellular membranes and additional host factors [6], [7], [8], [9]. The specific membrane alteration induced during HCV RNA replication has been designated as membranous web [10], [11]. Nonstructural proteins 3 through 5B are essential for HCV RNA replication, and their functional complex is referred to as replicase.

Nonstructural protein 4B (NS4B) is the least characterized HCV protein. However, evidence from biochemical, structural and genetic studies as well as electron microscopy (EM) indicates that NS4B is a key organizer of HCV replication complex formation (reviewed in [12]). Indeed, NS4B has been shown to induce formation of the membranous web which serves as a scaffold for the viral replicase [10], [11]. More recent work has shown that the other nonstructural proteins, especially NS5A, contribute to the formation of double membrane vesicles (DMVs) which make up the membranous web [13] and are believed to represent sites of HCV RNA replication [14].

NS4B is a 27-kDa integral membrane protein comprising an N-terminal part (amino acids [aa] 1 to ∼69), a central part harboring four predicted transmembrane segments (aa ∼70 to ∼190), and a C-terminal part (aa ∼191 to 261). The N-terminal part comprises a predicted and a structurally resolved amphipathic α-helix, designated as AH1 and AH2, respectively. AH2 comprises aa 42–66 and has been shown to play an important role in HCV RNA replication [15]. Intriguingly, it has the potential to traverse the phospholipid bilayer as a transmembrane segment, likely upon oligomerization [15], [16], [17], [18]. Hence, the N-terminal part of NS4B may adopt a dual cytosolic and ER luminal topology. However, this has not been explored in a functional, replicative context.

AH1 was predicted as an amphipathic α-helix and reported to mediate membrane association and HCV RNA replication [19]. However, membrane association of AH1 is debated [12] and the actual structure as well as detailed functional analyses, covering the complete HCV life cycle, have not been reported.

Here, we describe the three-dimensional structure of AH1 and provide a detailed structure-function analysis, indicating that this structurally highly conserved segment of NS4B possesses a dual role in HCV RNA replication and virus production. In addition, we demonstrate that the N-terminal part of NS4B adopts a dual membrane topology in an authentic replication context, allowing to speculate that the different functions of NS4B may be governed by different topologies.

## Results

### Sequence analyses and structure predictions

**Figure 1:**
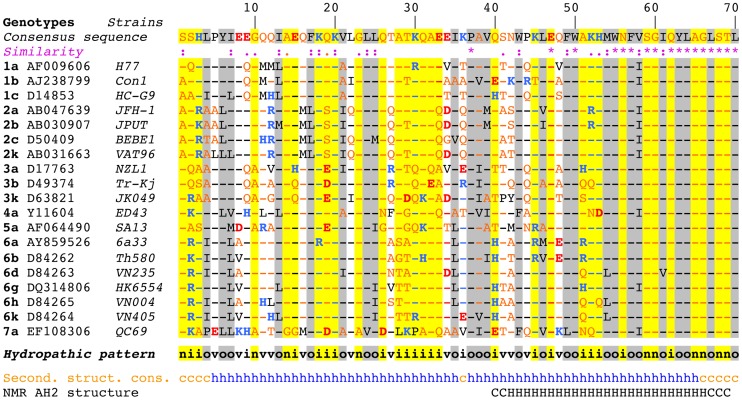
Sequence analysis of the N-terminal part of HCV NS4B. Multiple alignment of NS4B amino acid (aa) 1–70 sequences from representative HCV strains of confirmed genotypes [66] are shown (http://euhcvdb.ibcp.fr; [41]). Genotype, GenBank accession number, and strain are indicated for each sequence. Amino acids are numbered with respect to NS4B (top row). The consensus sequence (top row) was deduced from the ClustalW multiple alignment of the indicated NS4B sequences [43]. To highlight the aa variability at each position, aa identical to the consensus sequence are indicated by hyphens. The degree of aa physicochemical conservation at each position can be inferred from the similarity index according to ClustalW convention (asterisk, invariant; colon, highly similar; dot, similar) [43] and the consensus hydropathic pattern: o, hydrophobic position (Pro, Val, Leu, Ile, Met, Phe, Tyr, Trp); n, neutral position (Gly, Ala, Ser, Thr); i, hydrophilic position (Asn, Gln, Asp, Glu, His, Lys, Arg); v, variable position (i.e. when both hydrophobic and hydrophilic residues are observed at a given position). To highlight the variable sequence positions in NS4B, conserved hydrophilic and hydrophobic positions are highlighted in yellow and gray, respectively. Residues are color-coded according to the Wimley and White hydrophobicity scales [67]: hydrophobic residues are black (Pro, Val, Leu, Ile, Met, Phe, Tyr, Trp); polar residues are orange (Gly, Ala, Ser, Thr, Asn and Gln); positively and negatively charged groups of basic (His, Lys, Arg) and acidic residues (Glu, Asp) are blue and red, respectively. Consensus secondary structure predictions of NS4B from representative HCV strains (Second. struct. cons.) are indicated as helical (h, blue) or undetermined (coil [c], orange). Predictions were made by using the web-based algorithms SOPM, HNNC, DSC, GOR IV, PHD, Predator and SIMPA96 available at the NPSA website (http://npsa-pbil.ibcp.fr; [42] and refs. therein). NMR AH2 structure (bottom row) denotes the conformation of residues determined previously by nuclear magnetic resonance (PDB entry 2JXF; [26]). Residue conformations are indicated as helical (H) or undetermined (C).

Sequence analyses and structure predictions were performed to assess the degree of conservation of the N-terminal part of NS4B and to identify potential structural determinants. The degree of aa conservation among different genotypes was investigated by ClustalW alignment of 27 reference sequences representative of the major HCV genotypes and subtypes. This alignment revealed that the segment comprising aa 50–70, including part of amphipathic α-helix AH2 (aa 42–69), is well conserved, whereas the aa 1–50 segment, including predicted amphipathic α-helix AH1, appears highly variable except for a few well-conserved positions (e.g., basic residues at positions 18 and 20 and a proline at position 38 (Fig. 1). However, the apparent variability is limited at most positions since the observed residues exhibit similar physicochemical properties, as indicated both by the similarity pattern (colons and dots) and the hydropathic pattern, where o, i, and n denote hydrophobic, hydrophilic, and neutral residues, respectively (see Legend to Figure 1 for details). Moreover, all secondary structure prediction methods indicate the presence of an α-helix in the segment comprising aa 5–35 in all genotypes. Hence, despite the apparent aa variability, conservation of the hydropathic pattern suggests that the overall structure of AH1 is conserved.

While AH2 was predicted and subsequently shown to associate with membranes, the N-terminal aa 1–40 segment does not show propensity to partition into a phospholipid bilayer [15]. This is in agreement with an analysis by Palomares-Jerez *et al.* [20] but in contrast to an earlier report by Elazar *et al.* [19] (see Discussion section).

### Structure of NS4B AH1

To gain insight into the structure and potential lipotropic properties of the NS4B aa 1–40 segment, the corresponding peptide derived from the HCV JFH1 strain (genotype 2a), designated as NS4B[1–40], was chemically synthesized, purified, and analyzed by circular dichroism (CD) and nuclear magnetic resonance (NMR).

**Figure 2:**
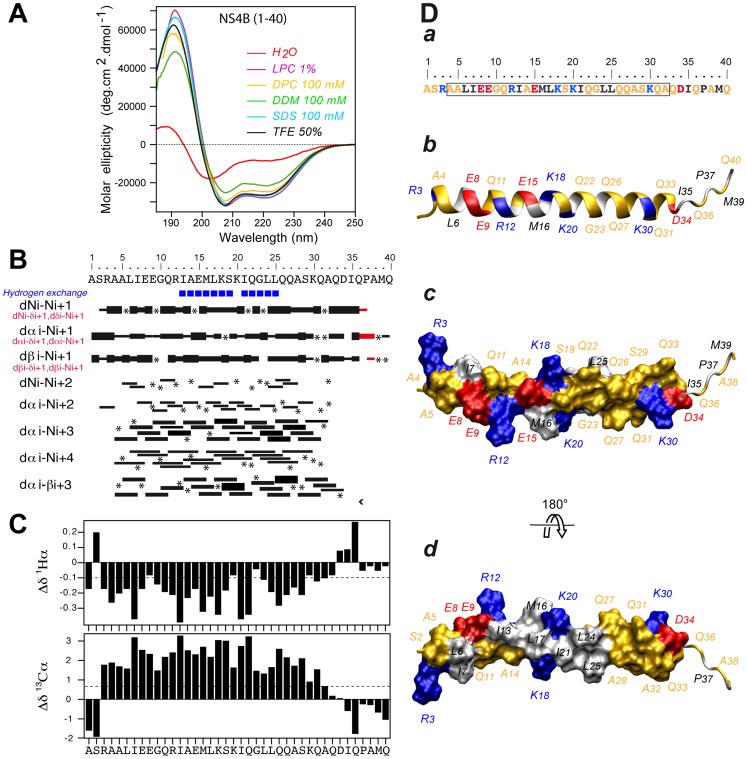
Structure of NS4B AH1. (**A**) Far UV circular dichroism (CD) spectra of synthetic peptide NS4B[1–40] recorded in 5 mM sodium phosphate pH 7.5 (H_2_O), complemented with either 50% 2,2,2-trifluoroethanol (TFE) or 1% L-α-lysophosphatidylcholine (LPC) or the following detergents: 100 mM sodium dodecyl sulfate (SDS), 100 mM n-dodecyl-β-D-maltoside (DDM), or 100 mM dodecylphosphocholine (DPC). (**B**) Summary of sequential (i, i+1) and medium-range (i, i+2 to i, i+4) nuclear Overhauser enhancements (NOEs) of NS4B[1–40] in 50% TFE. Sequential NOEs allowing the assignment of proline residues are indicated in red. Asterisks indicate that the presence of a NOE cross peak was not confirmed because of overlapping resonances or the lack of H assignment. Intensities of NOEs are indicated by the height of the bars. Amide protons that remained observable in nuclear magnetic resonance (NMR) spectra after three days in 50% D_2_0/50% deuterated TFE (TFE-*d_3_*) are indicated by blue squares (slow exchangeable protons). (**C**) NMR-derived ^1^Hα and ^13^Cα chemical shift differences were calculated by subtraction of the experimental values from the reported random coil conformation values in TFE [61]. The dashed lines indicate the standard threshold value of ΔHα (−0.1 ppm) or ΔCα (0.7 ppm) for an α-helix. (**D**) Representative NMR structure of NS4B[1–40] peptide in 50% TFE. The aa sequence of NS4B[1–40] is depicted in (*a*). The box indicates α-helix residues. Residues are color-coded as described in the Legend to Figure 1. (*b–d*) representative NMR structure model of NS4B[1–40] (PDB entry 2LVG) showing the amphipathic character of α-helix 4–32. *b*, ribbon representation of the side view colored as in panel *a*; *c* and *d*, hydrophilic side and hydrophobic side views of the molecular surface of amphipathic α-helix 4–32. Figures were generated from structure coordinates using VMD (http://www.ks.uiuc.edu/Research/vmd/) [68] and rendered with POV-Ray (http://www.povray.org/).

The secondary structure of NS4B[1–40] was examined by CD spectroscopy in various membrane mimetic media, including the lysolipid L-α-lysophosphatidylcholine (LPC), detergents (sodium dodecyl sulfate [SDS], n-dodecyl-β-D-maltoside [DDM], dodecylphosphocholine [DPC]), or co-solvents (2,2,2-trifluoroethanol [TFE]-water mixture) (Fig. 2A). In all cases, the CD spectra displayed the typical shape of an α-helix with two minima at 208 and 222 nm and one maximum around 192 nm. CD deconvolution indicated a similar α-helix content of 75±10%, although a weaker signal intensity was observed in the presence of DDM. These results indicate the high propensity of NS4B[1–40] to adopt an α-helical structure in a hydrophobic environment. Interestingly, the peptide solubilized in water displayed a complex spectrum with a weak maximum around 188 nm and two broad minima around 202 and 222 nm, indicating the presence of a mixture of α-helical and random structures. Accordingly, an α-helix content of approximately 22% together with 55% disordered structure was estimated with the various CD deconvolution methods used. Such a secondary structure content for a short peptide suggests that it possibly exists as soluble, micelle-like aggregates which stabilize some residual α-helical folding in aqueous solution through the formation of a hydrophobic core by the hydrophobic sides of several peptide monomers.

As samples of NS4B[1–40] prepared in deuterated micellar SDS and DPC displayed poorly resolved NMR spectra, the three-dimensional structure of the peptide was determined in 50% TFE-*d*2 which exhibits a CD spectrum comparable to those observed in LPC, SDS and DPC (Fig. 2A) and yielded well-resolved NMR spectra. Sequential assignment of all spin systems was completed and an overview of the sequential and medium-range nuclear Overhauser enhancement (NOE) connectivities is shown in Figure 2B. The NOE connectivity patterns demonstrated that the central part of the peptide, including residues 4–32, displays most characteristics of an α-helix, including strong dNN(i,i+1) and medium dαN(i,i+1) sequential connectivities as well as weak dαN(i,i+2), medium or strong dαN(i,i+3) and dαβ(i,i+3), as well as weak dαN(i,i+4) medium-range connectivities. Apart from this central α-helix, some rare connectivities of weaker intensity are present in both termini of the peptide as a sign of highly flexible unstructured ends. The NOE-based indications of an α-helical fold were supported by the deviation of the ^1^Hα and ^13^Cα chemical shifts from random coil values [21] (Fig. 2C). A series of continuous negative variations of ^1^Hα chemical shifts (Δδ^1^Hα <−0.1 ppm) and positive variations of ^13^Cα chemical shifts (Δδ^13^Cα>0.07 ppm) observed for residues 3 to 32 are indeed typical of an α-helical conformation. Based on the NOE-derived inter-proton distance and dihedral angle constraints deduced from chemical shifts, a set of 50 structures was calculated with X-PLOR, and a final set of 37 low-energy structures that fully satisfied the experimental NMR data were retained. The number and types of NOE constraints used for the structure calculations as well as the statistics for this final set of 37 structures are provided in Table S1. All structures show a regular α-helical conformation extending from residues 4 to 32. A superimposition of the calculated structures is shown in Figure S1. As illustrated in Figure 2D for the representative structure of NS4B[1–40], the central part of the α-helix, including residues Ile 13 to Leu 25, clearly exhibits an amphipathic character, with all hydrophobic residues exposed on one side and charged as well as polar residues on the opposite side. Interestingly, the α-helical folding of this central segment appears to be particularly stable, as indicated by the very slow amide proton exchange observed by NMR in this region (Fig. 2B, blue squares). Hence, this segment likely constitutes an important structural scaffold. In addition, the bulky hydrophobic residues Leu 6 and Ile 7 are also located on the hydrophobic side of the α-helix, suggesting that aa segment 6–25 may bind to a membrane hydrophobic core. These features suggest that AH1 may interact with the membrane interface in an in-plane topology, at least transiently. The C-terminal end (aa residues 26–32) of the α-helix does not include any hydrophobic residue but comprises an intriguingly large number of glutamine residues, which are, however, not conserved in all HCV genotypes (Fig. 1). Because of its purely hydrophilic nature, the folding of this C-terminal part most likely depends on interactions with other parts of NS4B and/or other interaction partners.

### NS4B AH1 from different hepaciviruses share conserved structural features

**Figure 3:**
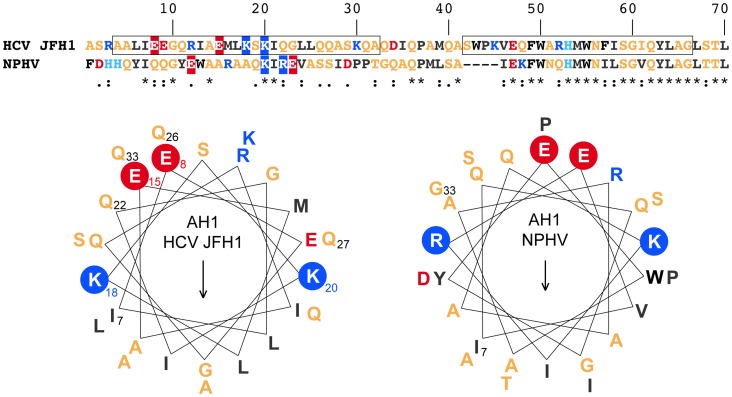
NS4B AH1 is structurally conserved among phylogenetically distant hepaciviruses. The sequences of NS4B amino acids (aa) 1–70 from the HCV JFH1 strain (genotype 2a; UniProtKB entry Q99IB8) and the non primate hepacivirus strain NPHV-B10-022 (UniProtKB entry I1ZAS8) were aligned by ClustalW (see Legend to Figure 1 for color code). The similarity index according to ClustalW convention is shown below (asterisk, invariant; colon, highly similar; dot, similar). Helical wheel projections of aa 7–33 of HCV JFH1 and NPHV-B10-022 are shown in the lower panel. The structurally conserved positively charged aa residues flanking the amphipathic α-helices (K, lysine; R, arginine) as well as the glutamate (E) residues on the hydrophilic side are highlighted.

To investigate the structural conservation of AH1, we compared two distantly related hepacivirus species, HCV and NPHV. As shown in the upper part of Figure 3, there is only a low degree of aa conservation between the NS4B aa 1–40 segments from the HCV JFH1 strain and NPHV-B10-022 [3]. However, the latter was strongly predicted to adopt an α-helical fold by secondary structure analyses performed as described in the Legend to Figure 1. Based on these observations, α-helix projections generated by Heliquest [22] were compared, as shown in the lower part of Figure 3. Interestingly, despite divergent primary aa sequences, α-helix projections showed a conservation of the amphipathic character of AH1 and of a number of structurally conserved features, including the presence of two positively charged residues, i.e. arginine or lysine, flanking the helices at the borders between the hydrophilic and hydrophobic sides as well as the presence of two negatively charged glutamate residues aligned on the hydrophilic side of AH1 from HCV and NPHV (Fig. 3). Taken together, these observations highlight structurally conserved features of AH1 from phylogenetically distant hepaciviruses, suggesting similar functions of this segment of NS4B within the *Hepacivirus* genus.

### The positively charged residues flanking AH1 are crucial for HCV RNA replication

**Figure 4:**
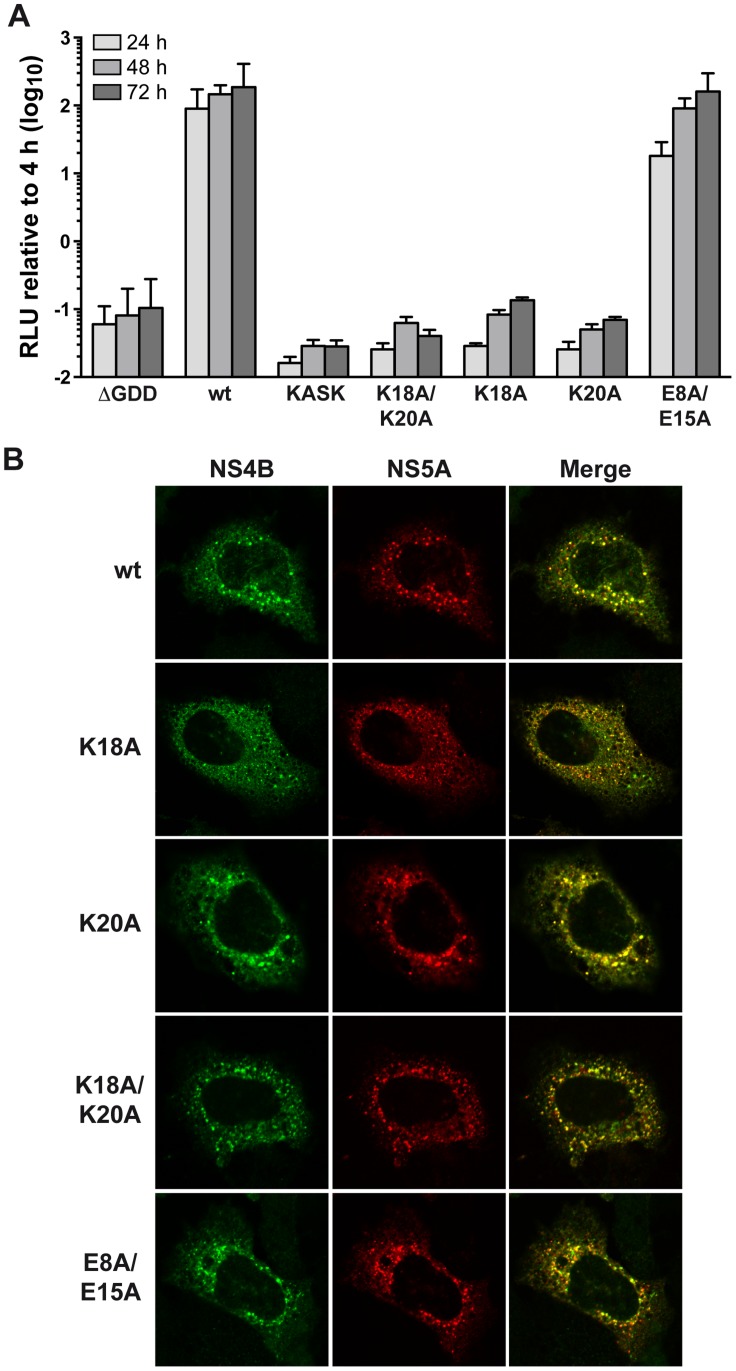
The conserved positively charged amino acids in NS4B AH1 are essential for RNA replication . (**A**) AH1 mutants introduced into a subgenomic replicon construct harboring a firefly luciferase reporter gene were analyzed in Huh-7.5 cells by luciferase activity measurement at 4, 24, 48 and 72 h post-electroporation of *in vitro* transcribed RNA. Relative light units (RLU) were normalized to values measured at 4 h. ΔGDD represents a replication-deficient control with an inactivating deletion in the RNA-dependent RNA polymerase active site. Results from a representative experiment performed in triplicate are shown. (**B**) Alanine substitution of the conserved positively charged amino acids in AH1 does not alter the subcellular localization of NS4B. T7 RNA polymerase-driven N3-5B polyprotein expression constructs harboring the different NS4B mutations were transfected into H7-T7-IZ cells which constitutively express the T7 polymerase, followed by immunofluorescence analyses as described in the Materials and Methods section. Polyclonal antibody #86 against NS4B and monoclonal antibody 9E10 against NS5A, were used as primary antibodies.

The role of the structurally conserved features of AH1 in HCV RNA replication was investigated by the use of a subgenomic JFH1 replicon harboring a luciferase reporter gene. As shown in Figure 4A, alanine substitution of Lys 18 and Lys 20, either simultaneously (K18A/K20A) or individually (K18A and K20A), abrogated HCV RNA replication, as inferred by comparison with the non-replicative ΔGDD polymerase mutant. In addition, insertion of one alanine residue between Lys 18 and Ser 19 (KASK), resulting in a 110° twist of the α-helix and thus an altered positioning of the two lysine residues in positions 18 and 20, abrogated HCV RNA replication. Hence, the positively charged residues flanking AH1 on either side are required for HCV RNA replication. By contrast, alanine substitution of the two conserved glutamate residues highlighted above (E8A/E15A) did only slightly affect RNA replication at an early time point (about 5-fold reduction in relative light units [RLU] at 24 h, but no appreciable difference at 48 and 72 h).

To explore the mechanism by which substitution of the positively charged aa residues affects HCV RNA replication, the subcellular localization of NS4B harboring the different mutations was investigated in Huh-7 cells using a T7 RNA polymerase-based NS3-5B polyprotein expression system [23]. As shown in Figure 4B, all mutants displayed a similar cytoplasmic, reticular and dot-like distribution pattern, as described previously for NS4B and corresponding to the typical localization on membranes of the ER and of ER-derived modified membranes making up the membranous web [11], [15]. In addition, all mutants colocalized with NS5A to the same extent as the wild-type. These observations indicate that the replication defect of mutants K18A/K20A, K18A and K20A cannot be explained by an aberrant subcellular localization of NS4B or an obvious decrease in NS4B-NS5A colocalization.

### NS4B AH1 contributes to proper DMV formation

**Figure 5:**
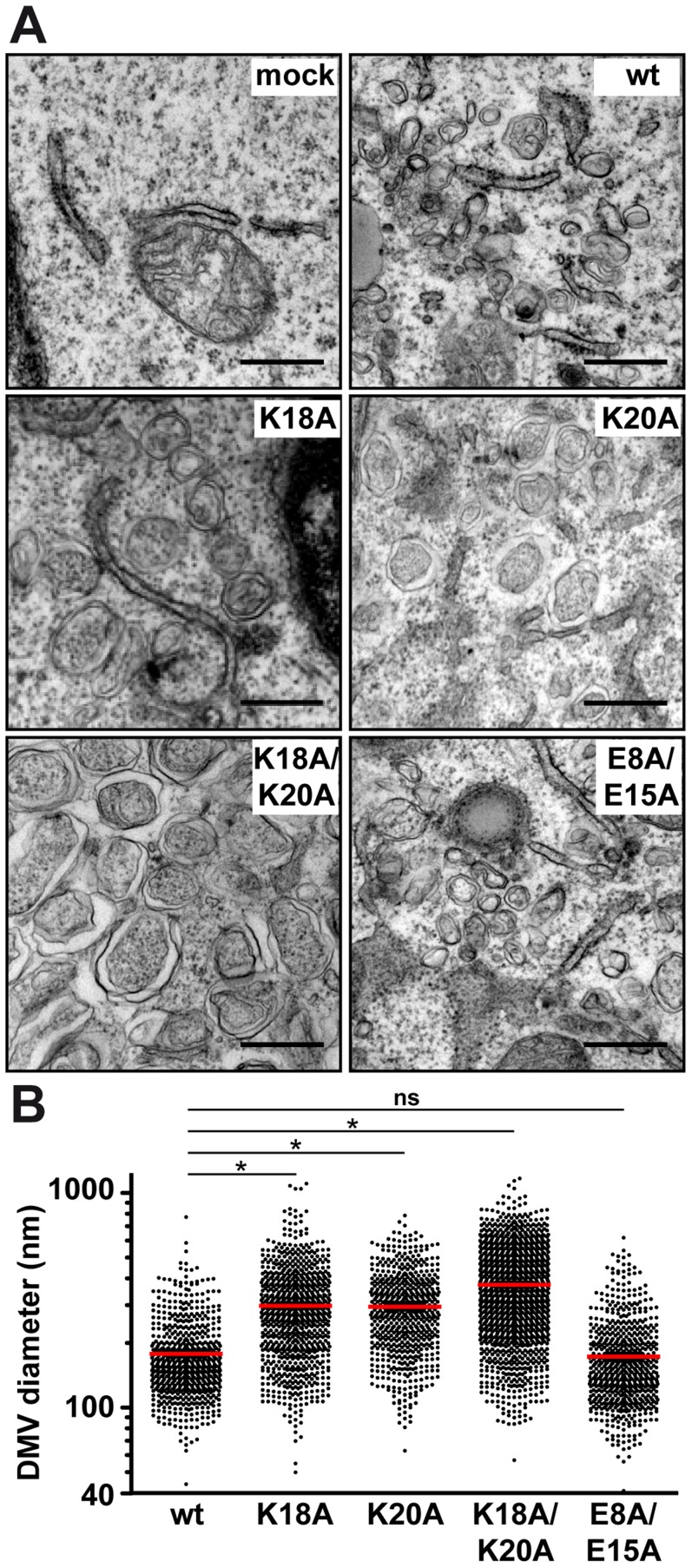
Mutations of the conserved positively charged amino acids in NS4B AH1 affect the morphology of double-membrane vesicles. (**A**) Electron microscopy (EM) analyses of H7-T7-IZ cells transfected with empty vector (mock) or T7 RNA polymerase-driven N3-5B polyprotein expression constructs harboring wild-type (wt) or mutant NS4B. Cells were fixed 24 h post-transfection and processed for EM as described in the Materials and Methods section. The scale bar corresponds to 500 nm. Note that double-membrane vesicles (DMV) induced by mutants K18A, K20A and K18A/K20A are larger as compared to wt and E8A/E15A. (**B**) Graphical representation of DMV diameter induced by wt and NS4B mutants. The analysis is based on at least 621 DMVs from at least 10 different transfected cells. Mean value ± SEM for wt = 178.4±3.1 nm (n = 666); K18A = 298.3±5.1 (n = 832); K20A = 295.1±5.0 (n = 621); K18A/K20A = 374.0±5.0 (n = 1317). Horizontal lines (red) indicate mean values. * P≤0.0001; ns, non significant (P>0.25).

To gain deeper insight into the consequences of removal of the positively charged residues flanking AH1, we investigated the ultrastructure of membrane rearrangements induced by the different NS4B mutants by EM. To this end, the mutants were expressed in Huh-7 cells using a T7 RNA polymerase-based NS3-5B polyprotein expression system, as above. Previous work had shown that DMVs are formed in this system and that these closely resemble the HCV-induced membrane rearrangements observed in the context of subgenomic RNA replication and HCV infection [13], [24]. As shown in Figure 5A, regular round-shaped DMVs were readily observed for mutants K18A/K20A, K18A and K20A but, strikingly, these exhibited a large increase in diameter as compared to the ones formed by the wild-type construct. By contrast, mutant E8A/E15A, which replicated almost as wild-type, formed DMVs that were indistinguishable from wild-type. Quantitation of the DMV diameter for each construct demonstrated that DMVs formed by mutants K18A/K20A, K18A and K20A were significantly larger than the ones formed by the wild-type as well as by mutant E8A/E15A (Fig. 5B; see Figure Legend for details). Taken together, these observations indicate that the loss of one of the conserved positively charged residues flanking AH1, i.e. Lys 18 and/or Lys 20, results in the formation of larger DMVs that do not support HCV RNA replication.

### Reverse genetics confirm a requirement for HCV RNA replication of positively charged residues flanking NS4B AH1

**Figure 6:**
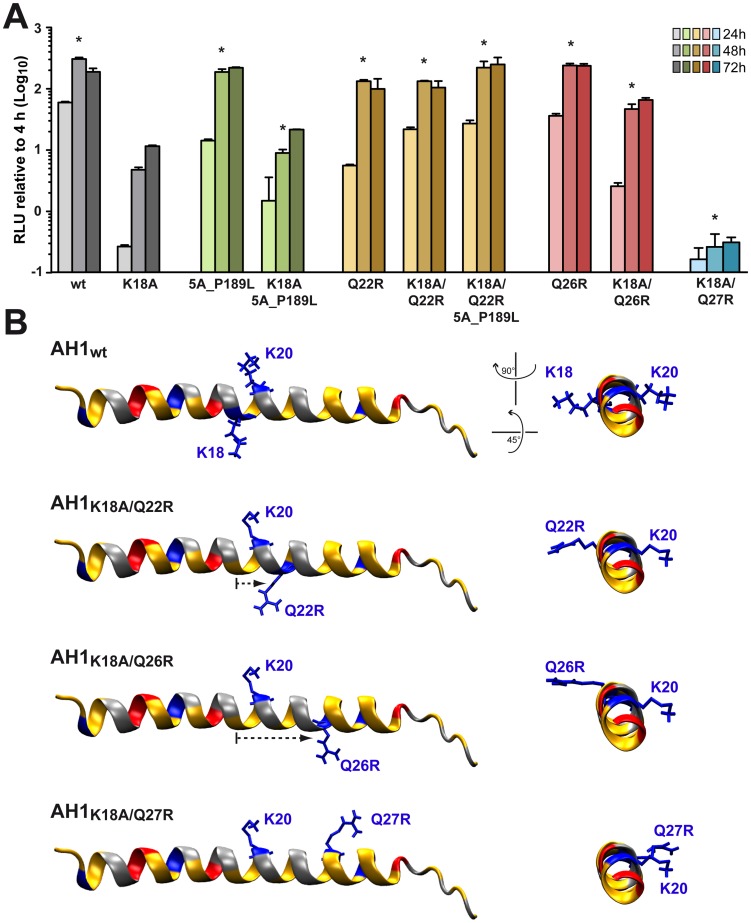
Analysis of pseudorevertants selected with NS4B AH1 mutant K18A. (**A**) Selected amino acid changes identified following transfection of a selectable subgenomic replicon harboring the NS4B AH1 K18A mutation (see Table 1) were reengineered into a subgenomic HCV replicon harboring a luciferase reporter gene. *In vitro* transcribed RNAs were electroporated into Huh-7.5 cells, followed by luciferase activity measurement at 4, 24, 48 and 72 h post-electroporation. Relative light units (RLU) were normalized to values measured at 4 h. Results from two independent experiments were pooled (n = 8 for each construct and time point). For statistical analyses, RLU measured at 48 h were compared to the 48-h values obtained with mutant K18A. *, P<0.001. (**B**) Structure models of the different AH1 sequences were established by using Swiss-PdbViewer 4.01 software [69] and drawn by using VMD 1.9 software [68].

In order to further assess the importance of the positively charged residues flanking AH1, mutations K18A, K20A and K18A/K20A were introduced into a selectable subgenomic JFH1 replicon harboring a neomycin resistance cassette in the first cistron. The corresponding *in vitro* transcribed RNAs were electroporated into Huh-7.5 cells, followed by selection with 500 µg/ml G418 for 3 weeks. About 70 G418-resistant colonies per µg of electroporated RNA were obtained for mutant K18A whereas mutants K18A/K20A and K20A did not yield any viable colonies. Total RNA was extracted from pooled colonies and the NS3-5B region amplified by RT-PCR, followed by cloning of amplicons and sequencing of 10 DNA subclones. Sequence analyses revealed that the K18A mutation in AH1 was retained in all clones. Amino acid changes identified elsewhere in the NS3-5B region are listed in Table 1. Strikingly, mutation P189L in domain I of NS5A was found in 9 out of 10 clones. The only clone which did not harbor this aa change in NS5A showed a substitution of Gln 26 by arginine (Q26R) in NS4B. Interestingly, this mutation results in a new positively charged residue flanking the hydrophobic side of AH1 (Fig. 3). Selection of a compensatory positively charged residue flanking AH1 was also observed in clones harboring pseudoreversions Q22R and Q27R, which coexisted with the NS5A P189L change. The relevance of these compensatory mutations was tested by reintroducing them, alone or in combination with K18A, into a JFH1 subgenomic replicon harboring a luciferase reporter gene. As shown in Figure 6A, the NS5A P189L change alone did not confer any replication advantage to the wild-type replicon and did only slightly (about 2-fold) improve replication of the NS4B K18A mutant. However, the Q22R and Q26R changes in NS4B AH1 strongly enhanced RNA replication capacity of mutant K18A (about 1.5 log for Q22R and 1.0 log for Q26R). Remarkably, the addition of NS5A change P189L to the NS4B K18A/Q22R mutant completely rescued RNA replication, thereby supporting the notion of a functional interaction between NS4B AH1 and NS5A. Finally, the NS4B Q27R change, identified in one clone harboring a number of additional aa changes (Table 1), did not rescue RNA replication of mutant K18A.

**Table 1:** Pseudoreversions selected with the NS4B K18A mutant.

Clone n°	NS3	NS4B^1^	NS5A
**#1** 2	T40I		N62D
			P189L
**#2**		*Q26R*	
**#3**		*Q22R*	P189L
**#4**		P44L	P189L
**#5**	Y57H	R214K	P189L
**#6**	R25H		P189L
			L259F
**#7**		M39L	P189L
**#8**			P189L
**#9**		*Q27R*	K30T
			K166R
			P189L

1 Mutations indicated in italic reside in AH1.

2 Clone #1 has been identified twice by sequencing.

Interestingly, modeling of pseudorevertants Q22R and Q26R in the NMR structure of AH1 revealed that the lateral chains of the two selected arginine residues are oriented to the same helix side as the mutated lysine residue in position 18, opposite to Lys 20, thereby restoring a positively charged residue flanking AH1 at distances of one (Q22R) or two (Q26R) α-helix turns (Fig. 6B). By contrast, the Q27R change, which was unable to rescue the replication defect of mutant K18A when reintroduced alone, results in a positively charged residue oriented to the opposite side of Lys 18, i.e. to the same side as Lys 20, thereby failing to restore a conserved feature of AH1. Taken together, selection of pseudoreversions confirms the requirement for two positively charged residues flanking AH1 on either side, as initially suggested by their conservation throughout the different HCV genotypes as well as the *Hepacivirus* genus (Fig. 3).

### NS4B AH1 is involved in virus production

**Figure 7:**
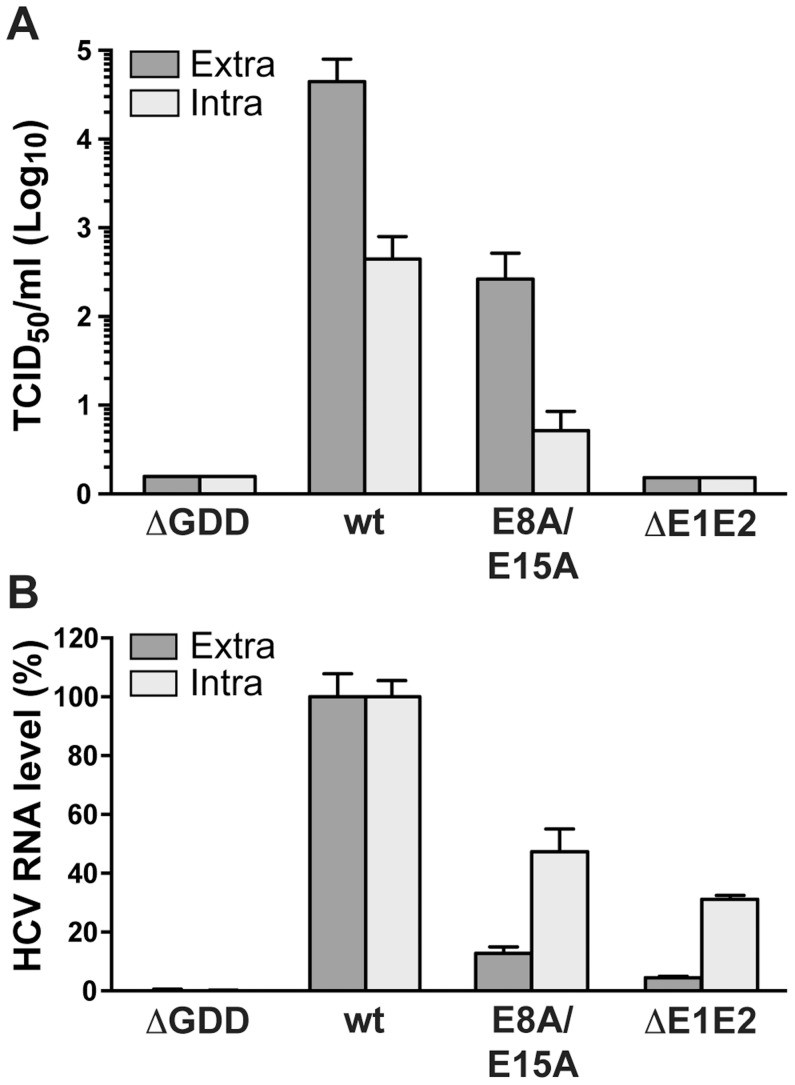
Alanine substitution of the conserved acidic residues on the hydrophilic side of NS4B AH1 affects virus production. (**A**) Extra- and intracellular infectivity after electroporation of a replication-deficient (ΔGDD), wild-type (wt), E8A/E15A mutant and envelope glycoprotein-deficient (ΔE1E2) full-length Jc1 genomes was determined by 50% tissue culture infective dose (TCID_50_) measurement at 48 h post-electroporation. (**B**) Intra- and extracellular HCV RNA levels were determined by RT-PCR 48 h post-electroporation and normalized to GAPDH mRNA level. Results measured for the wt were set to 100%. Note the use of a linear scale as opposed to the logarithmic scale used in panel A.

As mutant E8A/E15A did not show any significant replication defect, we introduced these aa substitutions into a full-length Jc1 (J6/JFH1 chimeric) HCV genome and assessed virus production by 50% tissue culture infective dose (TCID_50_) determination. As shown in Figure 7A, infectious virus production by this mutant was strongly reduced, with more than 100-fold lower extra- and intracellular TCID_50_ yields as compared to the wild-type virus. These results clearly indicate that NS4B AH1 possesses a role in HCV particle production. Quantitation of intra- *vs*. extracellular TCID_50_ suggests that the defect is primarily at the level of particle assembly, but an effect on release cannot be excluded (Fig. 7A). Quantitation of intra- *vs*. extracellular HCV RNA levels demonstrates that mutant E8A/E15A replicates in a full-length viral genome context and confirms the selective defect in virus production of this mutant (Fig. 7B).

### The N terminus of NS4B exhibits a dual topology in a replicative context

As discussed in the Introduction section, the N terminus of NS4B has previously been proposed to adopt a dual topology, based on evidence from glycosylation acceptor site tagging experiments performed in an *in vitro* translation system as well as in transiently transfected mammalian cells [16], [17]. In addition, we had shown that AH2 has the potential to traverse the phospholipid bilayer and that oligomerization of AH2 is likely required for this process [15], [18]. However, the membrane topology of the N-terminal part of NS4B, comprising AH1 and AH2, has not been investigated in a functional, replicative context. Hence, we took advantage of a recently developed JFH1 subgenomic replicon harboring an HA epitope tag insertion after NS4B aa position 38, i.e. between AH1 and AH2 [14], and examined the topology of the epitope tag by selective membrane permeabilization and immunofluorescence analyses.

**Figure 8:**
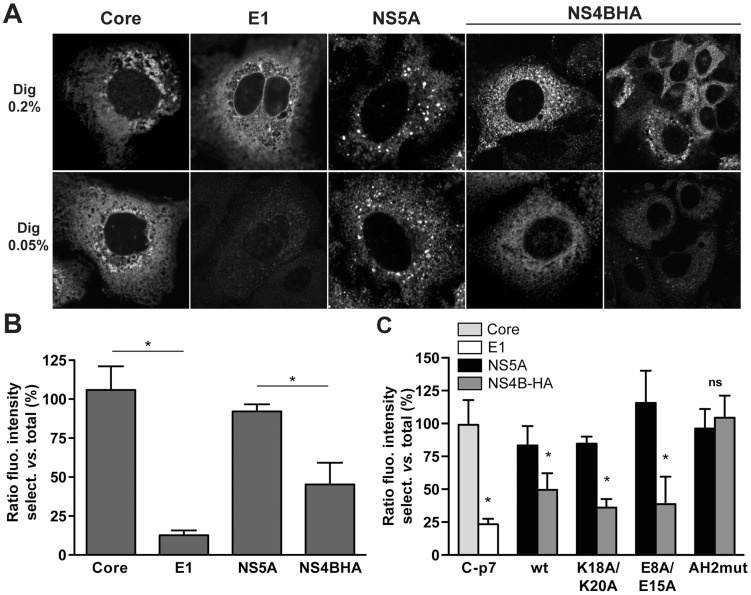
The N-terminal part of NS4B assumes a dual topology in a replicative context. (**A**) Huh7-Lunet cells harboring a subgenomic HCV JFH1 replicon with an HA tag inserted between NS4B AH1 and AH2 (see Materials and Methods section) were subjected to total (0.2% digitonin [Dig 0.2%], upper row) or selective membrane permeabilization (0.05% digitonin [Dig 0.05%], lower row), as described in the Materials and Methods section. As a control, replicon cells were transfected with a CMV promotor-driven core-E1-E2-p7 expression construct. Core and NS5A served as controls for cytosolically oriented proteins, E1 as a control for a luminally oriented protein. Monoclonal antibodies C7-50 against HCV core, A4 against E1, 9E10 against NS5A, and HA-7 against the HA tag were used, followed by anti-mouse IgG Alexa Fluor 488 as secondary antibody. Images were acquired on a confocal laser scanning microscope with the same settings for each antibody and condition. Analogous results were obtained when polyclonal antibody Y-11 against the HA tag was used instead of monoclonal antibody HA-7. (**B**) Histogram of the fluorescence intensity ratios between selective and total membrane permeabilization conditions. Fluorescence intensities in 10–60 images for each condition were determined by using ImageJ software [70]. (**C**) H7-T7-IZ cells were transfected with a CMV promotor-driven core-E1-E2-p7 expression construct or T7 RNA polymerase-driven N3-5B polyprotein expression constructs harboring the different mutations in HA-tagged NS4B, followed by selective membrane permeabilization and immunofluorescence microscopy using the same antibodies as in panel A. Fluorescence intensity ratios between selective and total membrane permeabilization conditions were determined as in panel B. AH2mut is a previously described mutant harboring alanine substitution of 6 aromatic amino acid residues in NS4B AH2 [15]. This mutant is unable to translocate AH2 through the membrane bilayer. * P≤0.0001; ns, non significant (P>0.25).

In a series of preliminary experiments, we found that incubation of fixed cells with 0.05% digitonin for 15 min at 4°C allowed for selective permeabilization of the plasma membrane but not the endoplasmic reticulum (ER) membrane of Huh7-Lunet cells which are highly permissive for HCV replication [25]. By contrast, 0.2% digitonin under the same experimental conditions permeabilized both membrane compartments. As shown in Figure 8A, the cytosolically oriented core and NS5A proteins could be detected at the same fluorescence intensity under both selective and total permeabilization conditions while the ER luminally oriented E1 glycoprotein could be detected only after total membrane permeabilization. Interestingly, the HA tag inserted between NS4B AH1 and AH2 was consistently detected at an approximately 50% reduced fluorescence intensity under selective as opposed to total membrane permeabilization conditions (Fig. 8A and 8B). These results, which were independent from the choice of anti-HA antibody, indicate a dual topology of the HA tag and, thereby, of the N-terminal part of NS4B in a functional, replicative context.

To investigate whether the AH1 mutations described and characterized above affect the membrane topology of the N-terminal part of NS4B, we introduced mutations K18A/K20A and E8A/E15A into a T7 RNA polymerase-driven construct allowing the expression of NS3-5B including NS4B harboring the HA tag between AH1 and AH2. As shown in Figure 8C and Figure S2, and consistent with the results obtained in a replicative context, the fluorescence intensity for HA tag detection was reduced by about 50% under selective as compared to total membrane permeabilization conditions. Mutations K18A/K20A and E8A/E15A did not affect this ratio. By contrast, a previously described AH2 mutant which is unable to associate with membranes, oligomerize and traverse the phospholipid bilayer, designated as AH2mut [15], [18], was detected at equal fluorescence intensity under both selective and total permeabilization conditions. This additional control validates our selective permeabilization analyses, confirming that the N-terminal part of NS4B adopts a dual topology in an authentic replication context as well as in the context of heterologous NS3-5B expression and is unaffected by mutations K18A/K20A and E8A/E15A which abrogate HCV RNA replication and strongly reduce virus production, respectively.

## Discussion

In the present study, we report the three-dimensional structure of NS4B AH1 and provide a detailed structure-function analysis of this N-terminal amphipathic α-helix. We show that AH1 folds as an α-helix extending from aa 4 to 32 and propose that the fold as well as a number of key structural features are conserved across hepaciviruses. Site-directed mutagenesis and reverse genetics revealed that the conserved positively charged aa residues 18 and 20 flanking AH1 on either helix side are essential for HCV RNA replication. EM analyses demonstrated that these residues play a crucial role in determining the correct size of the DMVs making up the membranous web. Furthermore, the conserved acidic residues 8 and 15 on the hydrophilic side of AH1 were found to be involved in virus production, likely at the level of assembly. Hence, NS4B AH1 possesses a dual role in HCV RNA replication and virus production. Finally, an HCV replicon harboring an epitope tag between AH1 and AH2 allowed to study the topology of the N-terminal part of NS4B in a functional context. Our findings obtained by selective membrane permeabilization and immunofluorescence analyses indicate that this part of NS4B adopts a dual membrane topology in an authentic replication context, likely determined by partial translocation of AH2 across the membrane.

Although AH1 of NS4B is globally amphipathic, NMR analyses revealed that the central amphipathic region comprising aa 13–25 is particularly stable and likely constitutes an important structural scaffold. Together with N-terminal hydrophobic residues Leu 6 and Ile 7, it might interact with the membrane interface in an in-plane topology, at least transiently. Such a binding might also allow stabilization of the α-helical fold of the hydrophilic C-terminal part comprising aa 26–32 upon interaction with the membrane. However, the highly hydrophilic character of this latter part suggests that it may adopt alternative conformations, possibly upon interaction with other parts of NS4B and/or other interaction partners. In addition, the overall hydrophilic character of AH1 is compatible with the absence of direct membrane association *per se*, as reported previously [15], and suggests that this α-helix may be involved in alternative intra- and/or inter-molecular interactions, e.g. with NS5A (see below).

The present work complements the structures of NS4B AH2 [15], H1 (RM and FP, unpublished) and H2 [26]. AH2 represents an amphipathic α-helix spanning aa 42 to 66 and H2 a “twisted” amphipathic α-helix extending from aa 229 to 253. Although AH1, AH2 and H2 share amphipathic character and play critical roles in HCV RNA replication, their structures are very different. AH1 is mainly hydrophilic, possesses a limited hydrophobic side lacking aromatic residues and is flanked by conserved positively charged residues, AH2 has a very hydrophobic side including 6 highly conserved aromatic residues, and H2 represents a “twisted” amphipathic α-helix. Future work will have to address the structure of the central part of NS4B believed to harbor four transmembrane segments and the ultimate goal will be to solve a complete structure of this integral membrane protein.

Our mutagenesis analyses show that AH1 contributes to RNA replication by affecting the biogenesis of DMVs making up the membranous web. While we previously did not observe a direct interaction of AH1 with cellular membranes [15], it is likely that AH1 interacts with membranes when brought into the appropriate context, e.g. in the presence of AH2 and/or upon oligomerization of NS4B. AH1 may thereby influence membrane curvature induction, resulting in proper DMV formation and assembly of a functional replication complex. Indeed, membrane-associated amphipathic α-helices flanked by positively charged residues have been described to play a role in membrane curvature sensing [27] (reviewed in [28]). As an example, Nath *et al.* recently demonstrated that the autophagy regulator Atg3 possess an N-terminal amphipathic α-helix which serves as membrane curvature sensor [29]. Comparably to AH1, the Atg3 amphipathic α-helix possesses two lysine residues (aa positions 9 and 11) bordering a hydrophobic face devoid of aromatic aa residues. As we observed DMVs of larger diameter in the AH1 mutant lacking at least one lysine, we may hypothesize that NS4B AH1 plays a similar role in membrane curvature sensing and induction. Hence, AH1 may interact with the surface of a “pre-curved” membrane (large DMVs), possibly induced by AH2 or another mechanism such as NS4B oligomerization, and then further bend the membrane to end up with smaller diameter DMVs competent for RNA replication. Supporting this hypothesis, disruption of the hydrophobic face by introduction of charged residues, similar to the Atg3 mutants described by Nath *et al.* [29], has previously been shown to abrogate RNA replication [19].

The size and morphology of DMVs have been reported to affect the replication of HCV and other positive-strand RNA viruses. In this context, pharmacologic inhibition or silencing of phosphatidylinositol-4 kinase IIIα or of its effector oxysterol-binding protein, both of which are required for HCV RNA replication, have been shown to reduce DMV diameter [30], [31]. A direct correlation between altered DMV morphology and impaired RNA replication has been demonstrated for mutations in the C-terminal part of NS4B [24]. Mutations in murine hepatitis virus nonstructural protein 4 have been reported to affect DMV morphology and RNA replication [32] and mutations in equine arteritis virus nonstructural protein 3 have been shown to impair DMV formation [33]. However, HCV mutants that increase the diameter of DMVs have to our knowledge not been described previously. Taken together, our observations and previous reports indicate that a defined size and morphology of DMVs is required for efficient viral RNA replication.

Selection for pseudoreversions and modeling in the three-dimensional structure revealed that the two positively charged residues flanking AH1 have to be located on opposite sides of the α-helix for NS4B to be functional in HCV RNA replication. It is possible that these residues stabilize electrostatic interactions with the negatively charged phospholipid head groups in-plane of a membrane surface. Analysis of pseudorevertants indicates that laterally oriented positively charged side chains on either side of the α-helix are required irrespective of their position along the helix. However, the fact that the two lysine residues in aa positions 18 and 20 are conserved across all HCV isolates indicates that viral fitness favors the presence of the positively charged residues at these two specific positions. In keeping with our observations, a mutagenesis study performed previously in a subgenomic replicon derived from the Con1 strain (genotype 1b) identified an important role for Lys 20, with pseudoreversion to positively charged aa residues, i.e. lysine or arginine, at positions 15 or 16 [34].

Selection of aa changes in NS5A, some of which we show here to partially rescue the defect of NS4B mutant K18A, point toward functional interactions and a concerted action of NS4B and NS5A in replication complex formation, as supported by recent EM analyses of the membrane rearrangements induced by NS4B and NS5A [13] as well as earlier work on the phosphorylation of NS5A [35], [36]. In our study, we have identified an NS5A P189L change in 9 out of 10 sequenced clones. This aa change is located in domain I of NS5A, in a surface-exposed position that has the potential to interact with cytosolically oriented NS4B residue Lys 18 (not illustrated). However, it confers only a minor replication advantage in the NS4B K18A mutant context. Similarly, in a previous mutagenesis analysis of the C-terminal region of NS4B, the NS5A K139E change has been identified in several clones without conferring a major advantage for RNA replication on its own but partially required in combination with other pseudoreversions in NS4B [24]. Further investigating the interaction between NS4B and NS5A will in all likelihood reveal important insight into the roles of NS4B in HCV RNA replication and likely also virus production.

While the best known function of NS4B is its role in inducing the membrane rearrangements required for HCV RNA replication, there is growing evidence that NS4B is also involved in virus production [24],[35],[36]. The NS4B mutants described so far either enhanced virus assembly [24], [35] or decreased RNA encapsidation [36] and were all localized in the C-terminal region of NS4B. E8A/E15A is one of the first mutants reported to strongly decrease virus production with almost unimpaired HCV RNA replication capacity. Since intracellular virus titers are reduced to the same extent as extracellular titers, assembly is likely affected in this mutant. Given the evidence for functional interactions between NS4B and NS5A as well as the critical role of NS5A in virus assembly [37], [38], we may hypothesize that AH1 mutant E8A/E15A affects NS4B-NS5A interplay, thereby influencing virion assembly. Future work investigating the role of NS4B in virus production would be facilitated by the development of a complementation system that allows to separate the functions of NS4B in RNA replication and virus production.

**Figure 9:**
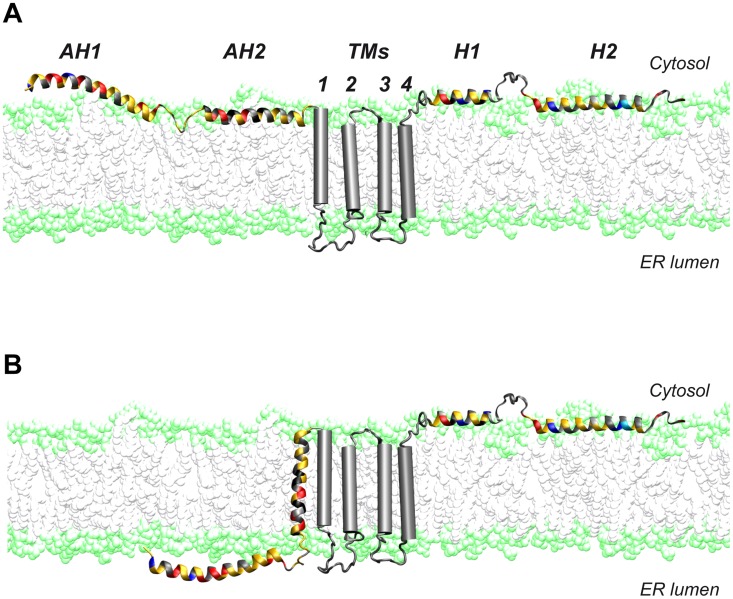
Dual membrane topology of HCV NS4B. The N-terminal part of NS4B assumes a dual membrane topology. Cartoons illustrating the (**A**) cytosolic and the (**B**) luminal endoplasmic reticulum (ER) membrane topology of the N-terminal part of NS4B are shown. TMs, predicted transmembrane segments.

A dual topology of the N terminus of NS4B has been suggested earlier [16], [17]. In line with these observations, we had previously shown that AH2 has the potential to traverse the phospholipid bilayer, likely upon oligomerization [15], [18]. Here, we provide evidence for a dual topology of the N-terminal part of NS4B in a functional, replicative context. A working model for these two topologies is illustrated in Figure 9. Based on its physicochemical properties, we could envision a scenario where AH1 remains in the ER lumen, possibly associated with the inner side of the ER membrane, sensing its curvature, and bending the membrane during membranous web formation. However, we believe that it is unlikely that AH1 itself traverses the membrane so that an ER luminal loop be formed between AH1 and AH2 (scenario therefore not illustrated in Fig. 9). Indeed, AH1 appears not hydrophobic enough to achieve a transmembrane topology and there is no obvious interaction platform between multiple copies of AH1 or AH1 and AH2 to yield a transmembrane hydrophobic complex.

Given the limited coding capacity of viral genomes, many encoded proteins have evolved to exert multiple functions. One strategy to achieve multifunctionality may be topological changes with respect of the membrane. In this context, the hepatitis B virus large surface protein and the fusion protein of Newcastle disease virus have been shown to adopt dual membrane topologies with potentially different functions [39], [40]. Based on these observations, it is tempting to speculate that the different topologies of NS4B may serve distinct functions in the HCV life cycle. Clearly, future work will have to address this intriguing possibility.

## Materials and Methods

### Sequence analyses and structure predictions

Sequence analyses were performed using the web-based tools available at the European HCV Database (http://euhcvdb.ibcp.fr/) [41] and the Network Protein Sequence Analysis (NPSA) website of the Institut de Biologie et Chimie des Protéines (IBCP) (http://npsa-pbil.ibcp.fr) [42]. Multiple sequence alignment and analyses of aa conservation were carried out with the ClustalW program using default parameters [43]. HeliQuest was used for α-helix projections (www.heliquest.ipmc.cnrs.fr) [22].

### Cell lines and reagents

Human hepatocellular carcinoma cell lines Huh-7.5 [44] (kindly provided by Charles M. Rice, The Rockefeller University, New York, NY) and Huh7-Lunet [25] were maintained in Dulbecco's modified Eagle medium supplemented with 10% fetal bovine serum. The Huh-7-derived cell line H7-T7-IZ, which stably expresses the T7 RNA polymerase, was maintained in the same medium supplemented with 5 µg/ml zeocin [13]. Transfections were performed by the use of polyethylenimine [45]. Monoclonal antibodies (mAbs) C7-50 against HCV core [46], A4 against HCV E1 [47] (kindly provided by Jean Dubuisson, University of Lille, France), as well as 9E10 [48] against HCV NS5A (kindly provided by Charles M. Rice) have been described. MAb AC-15 against β-actin was from Sigma-Aldrich. MAb HA-7 and polyclonal antibody Y-11 against the HA tag were from Sigma-Aldrich and Santa Cruz, respectively. Polyclonal antibody #86 against HCV NS4B has been described [37]. Secondary antibodies were anti-mouse-HRP (GE Healthcare), anti-rabbit-HRP (DAKO), Alexa Fluor 488- and Alexa Fluor 594-conjugated anti-mouse IgG, as well as Alexa Fluor 488-conjugated anti-rabbit IgG (Life Technologies).

### Plasmids

Plasmids pUHD15-1 (allowing expression of the tetracycline-regulated transactivator tTA) [49] and pUHD-Cp7con (allowing expression from a tTA-responsive promotor) were cotransfected to express core-E1-E2-p7 derived from the HCV H77 (genotype 1a) consensus clone [50], as described [51].

pFK-based plasmids pFK_Jc1_δg, pFK_Jc1ΔE1E2_δg, pFK-i389-neo-sg-JFH1, pFK_i389LucNS3-3′-NS4B^HA^31R_JFH_δg, pFK_i389LucNS3-3′_JFH_δg and the non-replicating control construct pFK_i389LucNS3-3′_NS5BΔGDD_JFH_δg have been described [14], [52], [53]. Plasmid pTM-NS3-3′_JFH allows for the T7 RNA polymerase-based expression of the HCV replicase proteins (NS3 to NS5B) [23].

Subgenomic HCV JFH1 replicon constructs harboring a bicistronic firefly luciferase reporter gene were based on pFK_i389LucNS3-3′_JFH_δg. Mutations K18A, K20A, K18A/K20A, KASK and E8A/E15A were generated by two-step PCR amplification using primers JFH1-5042-fd and AH1-K18A-rv, AH1-K20A-rv, AH1-KKAA-rv, AH1-KASK-rv or AH1-EEAA-rv, respectively (Table S2), followed by primers AH1-K18A-fd, AH1-K20A-fd, AH1-KKAA-fd, AH1-KASK-fd or AH1-EEAA-fd, respectively, and JFH1-7730-rv (Table S2). Final products were amplified by overlap extension PCR using primers JFH1-5042-fd and JFH1-7730-rv (Table S2), followed by cloning into the *Nsi*I-*Rsr*II sites of pFK_i389LucNS3-3′_JFH_δg, yielding constructs Luc-JFH1-AH1_K18A, Luc-JFH1-AH1_K20A, Luc-JFH1-AH1_K18A/K20A, Luc-JFH1-AH1_KASK and Luc-JFH1-AH1_E8A/E15A.

Neomycin-selectable subgenomic HCV JFH1 replicon constructs were generated by subcloning of the *Nsi*I/*Bsr*GI fragments from the Luc-JFH1 constructs above into pFK_i389-neo-sg-JFH1, yielding constructs Neo-JFH1-AH1_K18A, Neo-JFH1-AH1_K20A, Neo-JFH1-AH1_K18A/K20A and Neo-JFH1-AH1_KASK.

Pseudorevertant constructs were generated in subgenomic HCV JFH1 replicon constructs harboring a bicistronic firefly luciferase reporter gene by two-step PCR amplification using primers JFH1-5042-fd and JFH4BQ22R-rv or JFH4BQ26R-rv (Table S2) with either pFK_i389LucNS3-3′_JFH_δg (wild-type) or Luc-JFH1-AH1_K18A as template, followed by amplification of pFK_i389LucNS3-3′_JFH_δg (wild-type) or the corresponding sequence harboring the NS5A K189L mutation using primers JFH4BQ22R-fd or JFH4BQ26R-fd and JFH1-7730-rv (Table S2). Final products were amplified by overlap extension PCR using primers JFH1-5042-fd and JFH1-7730-rv (Table S2), followed by cloning into the *Nsi*I-*Rsr*II sites of pFK_i389LucNS3-3′_JFH_δg, yielding constructs Luc-JFH1-AH1_Q22R, Luc-JFH1-AH1_Q26R, Luc-JFH1-AH1_K18A/Q22R, Luc-JFH1-AH1_K18A-5A_K189L, Luc-JFH1-AH1_K18A/Q22R-5A_K189L and Luc-JFH1-AH1_K18A/Q26R. Construct Luc-JFH1-AH1_K18A/Q27R was generated by PCR using primers JFH1-5042-fd and JFH1-7730-rv (Table S2), followed by the same cloning strategy.

Jc1 full-length constructs were generated by subcloning of the *Avr*II/*Rsr*II fragments from the Luc-JFH1 constructs above into pFK-Jc1_δg, yielding constructs Jc1-AH1_E8A/E15A, Jc1-AH1_K18A, Jc1-AH1_K18A/Q22R, Jc1-AH1_Q26R and Jc1-AH1_K18A/Q26R. Construct Jc1-ΔGDD was generated by subcloning of the *Rsr*II/*Sfi*I from pFK_i389LucNS3-3′_NS5BΔGDD_JFH_δg into pFK-Jc1_δg.

pTM-based constructs harboring NS4B mutations were generated by subcloning of the *Nsi*I/*Rsr*II fragments from pFK_i389LucNS3-3′_JFH_δg or pFK_i389LucNS3-3′-NS4B^HA^31R_JFH_δg into pTM-NS3-3′, yielding constructs pTM-NS3-3′-AH1_K18A, pTM-NS3-3′-AH1_K20A, pTM-NS3-3′-AH1_K18A/K20A, pTM-NS3-3′-AH1_E8A/E15A, and pTM-NS3-3′-NS4B^HA^31R. Introduction of NS4B mutations into pTM-NS3-3′-NS4B^HA^31R was performed by two-step PCR using primers JFH1-5042-fd and AH1-KKAA-rv or AH1-EEAA-rv, followed by primers AH1-KKAA-fd or AH1-EEAA-fd and JFH1-7730-rv. Final overlap extension PCR was carried out with primers JFH1-5042-fd and JFH1-7730-rv, followed by cloning into the *Nsi*I-*Rsr*II sites of pTM-NS3-3′, yielding constructs pTM-NS3-3′-NS4B^HA^31R-AH1_K18A/K20A and pTM-NS3-3′-NS4B^HA^31R-AH1_E8A/E15A.

All contructs were verified by sequencing.

### *In vitro* transcription, electroporation, transient replication and infection assays

*In vitro* transcription of subgenomic replicon and full-length HCV RNA as well as electroporation were performed as described ([54] and references therein). RNA replication assay using a JFH1 subgenomic replicon harboring the firefly luciferase as reporter was performed as described previously [53], [55]. Jc1 virus was produced as described [56]. TCID_50_ was determined as described [48]. For the determination of intracellular infectivity, cells were harvested and subjected to three freeze and thaw cycles, followed by removal of debris by centrifugation for 2 min at 11,000× *g* before TCID_50_ determination.

### Immunoblot

Protein lysates were prepared and subjected to sodium dodecyl sulfate-polyacrylamide gel electrophoresis (SDS-PAGE) followed by immunoblot analysis as described previously [57].

### Peptide synthesis and purification

Peptide NS4B[1–40] from the HCV strain JFH-1 (accession number AB047639; aa sequence reported in Fig. 2) was synthesized on a Milligen 9050 apparatus, employing N-[9-fluorenyl]methoxycarbonyl (Fmoc) chemistry. The peptide was highly purified by reversed-phase high-performance liquid chromatography on a Nucleosil C18 column (120 Å, 5 µm, 250 mm) using a water-acetonitrile gradient containing 0.1% trifluoroacetic acid. The peptide was eluted as a single peak at 65% acetonitrile and identified by mass spectroscopy at its expected molecular mass.

### Circular dichroism

Far UV CD spectra were recorded on a Chirascan spectrometer (Applied Photophysics) calibrated with 1S-(+)-10-camphorsulfonic acid. Measurements were carried out at 298 K in a 0.1-cm path length quartz cuvette. Spectra were measured in a 180–260 nm wavelength range with an increment of 0.2 nm, bandpass of 0.5 nm and integration time of 1 s. Spectra were processed, baseline corrected, smoothed and converted with the Chirascan software. Spectral units were expressed as the mean molar ellipticity per residue by using the peptide concentration determined knowing the weighted NS4B[1–40] peptide used to prepare the NMR sample. Estimation of the secondary structure content was carried out on the DICHROWEB server (http://www.cryst.bbk.ac.uk/cdweb/) [58].

### NMR spectroscopy

Purified NS4B[1–40] peptide was dissolved in a mixture of 50% TFE-*d*_2_ (>99%) in H_2_O (v/v), and 2,2-dimethyl-2-silapentane-5-sulfonate was added to the NMR samples as an internal ^1^H chemical shift reference. Multidimensional experiments were performed at 25°C on a Bruker Avance 500 MHz spectrometer using standard homonuclear pulse sequences, including nuclear Overhauser enhancement spectroscopy (NOESY) (mixing times between 100 and 250 ms) and clean total correlation spectroscopy (TOCSY) (isotropic mixing time of 80 ms), as detailed previously [59], [60]. Water suppression was achieved by pre-saturation. Bruker Topspin software was used to process all data and Sparky was used for spectral analysis (http://www.cgl.ucsf.edu/home/sparky/). Intra-residue backbone resonances and aliphatic side chains were identified from homonuclear ^1^H TOCSY experiments and confirmed with ^1^H-^13^C heteronuclear single quantum correlation (HSQC) experiments in ^13^C natural abundance. Sequential assignments were determined by correlating intra-residue assignments with inter-residue cross peaks observed in bi-dimensional ^1^H NOESY. NMR-derived ^1^Hα and ^13^Cα chemical shifts are reported relative to the random coil chemical shifts in TFE [61].

### NMR-derived constraints and structure calculation

NOE intensities used as input for structure calculations were obtained from the NOESY spectrum recorded with a 150 ms mixing time and checked for spin diffusion on spectra recorded at lower mixing times (50 ms). NOEs were partitioned into three categories of intensities that were converted into distances ranging from a common lower limit of 1.8 Å to upper limits of 2.8, 3.9 and 5.0 Å, respectively. Protons without stereospecific assignments were treated as pseudoatoms, and the correction factors were added to the upper distance constraints [62]. Additionally, dihedral angle constraints calculated with Talos [63] from ^1^H and ^13^C chemical shifts were introduced. Three-dimensional structures were generated from NOE distances with the standard torsion angle molecular dynamics protocol in the XPLOR-NIH 2.30 program [64] using the standard force fields and default parameter sets. A set of 50 structures was initially calculated to widely sample the conformational space, and the structures of low energy with no distance restraint violations (>0.5 Å) were retained. The selected structures were compared by pairwise root-mean square deviation (RMSD) over the backbone atom coordinates (N, Cα and C′). Local analogies were analyzed by calculating the local RMSD of a tripeptide window sliding along the sequence. Statistical analyses, superimposition of structures and structural analyses were performed with MOLMOL [65] and the PDB Protein Structure Validation Suite.

### Selection for pseudorevertants, amplification of HCV RNA by RT-PCR, and sequence analyses

Huh-7.5 cells were electroporated with 1 µg *in vitro* transcribed RNA from neomycin-selectable subgenomic replicon constructs. Electroporated cells were resuspended in 10 ml of medium, followed by seeding of 10 µl, 100 µl or 1 ml into 10-cm tissue culture dishes. After 24 h, G418 was added at a concentration of 500 µg/ml and maintained until single cell clones became visible. Total RNA was extracted from pooled clones by using the RNeasy Mini Kit (Qiagen) according to the manufacturer's instructions. One µg total RNA was reverse transcribed with specific anchor primer JFH1-9442-rv, followed by PCR amplification using primers EMCV-fd and JFH1-9442-rv using PfuTurbo DNA Polymerase (Stratagene). Amplicons were cloned with Zero Blunt TOPO PCR Cloning Kit (Life Technologies), followed by sequencing of 10 clones.

### Quantitative RT-PCR

Glyceraldehyde 3-phosphate dehydrogenase (GAPDH) and HCV RNA levels were measured by SYBR green real-time PCR, as described [55].

### Selective permeabilization

H7-T7-IZ or Huh7-Lunet replicon cells were seeded onto glass coverslips. H7-T7-IZ cells were transfected 24 h later with pTM-NS3-3′ plasmids or with pUHD15-1 and pUHD-Cp7, allowing expression of the HCV core-p7 region. Twenty-four h post-transfection cells were fixed with 2% paraformaldehyde (Sigma-Aldrich) for 10 min and then permeabilized for 15 min at 4°C either by 0.2% or 0.05% digitonin (Sigma-Aldrich). Indirect immunofluorescence was performed as described previously [57]. Slides were viewed on a Leica SP5 confocal laser scanning microscope.

### Electron microscopy

Huh7-Lunet T7 cells were seeded onto glass coverslips. On the next day the cells were transfected with pTM-NS3-3′-based expression vectors by using the TransIT-LT1 transfection reagent (Mirus Bio). After 24 h the cells were washed three times with prewarmed PBS, fixed and processed for EM as described previously [24]. Specimens were examined with a Zeiss EM 10 transmission electron microscope at 60 kV.

### Statistical analyses

Significance values were calculated by using the unpaired t test with the GraphPad Prism 6 software package (GraphPad Software).

### Accession numbers

The atomic coordinates for the NMR structure of synthetic peptide NS4B[1–40] and the corresponding NMR restraints in 50% TFE are available in the Research Collaboratory for Structural Bioinformatics (RCSB) Protein Data Bank under accession number 2LVG (RCSB identification code 102881). The chemical shifts of NS4B[1–40] residues have been deposited in the BioMagResBank (BMRB) under the accession number 18568.

## Supporting Information

Table S1 **Statistics of final set of structures of NS4B[1–40]**.****
(DOCX)

Table S2 **Oligonucleotide sequences.**
(DOCX)

Figure S1 **Amino acid sequence and superimposition of the backbone heavy atoms (N, Cα, and C′) of the final set of 37 calculated structures for the best overlap of residues (PDB entry 2LVG).** This Figure shows that the α-helix is clearly defined between residues Ala 4 and Ala 32 (box), but with an RMSD of 2.1 Å (see Table S1).
(DOCX)

Figure S2 **H7-T7-IZ cells were transfected with T7 RNA polymerase-driven N3-5B polyprotein expression constructs harboring the indicated mutations in HA-tagged NS4B, followed by selective membrane permeabilization and immunofluorescence microscopy.** Cells were subjected to total (0.2% digitonin [Dig 0.2%], upper row) or selective membrane permeabilization (0.05% digitonin [Dig 0.05%], lower row), as described in the Materials and Methods section. Representative pictures for wild-type (wt) and the different mutants are shown.
(DOCX)
