# Supplementary material for: Aminoterminal Amphipathic α-Helix AH1 of Hepatitis C Virus Nonstructural Protein 4B Possesses a Dual Role in RNA Replication and Virus Production
Source: PLoS Pathog. 2014 Nov 13;10(11):e1004501. doi: 10.1371/journal.ppat.1004501 (PMC4231108; doi:10.1371/journal.ppat.1004501)
Supplement: Table S1 — Statistics of final set of structures of NS4B[1–40]. (DOCX) [file ppat.1004501.s001.docx]

**Supplementary Table 1. Statistics of final set of structures of NS4B[1-40].**

*A. Constraints used*

Distance restraints

Intra-residue 0

Total distance restraints 200

Dihedral angle constraints

Phi angles 34

Psi angles 34

*B. Statistics for the final X-PLOR structures*

Number of structures in the final set 37

X-PLOR energy (kcal.mol-1) -165.1 ± 13.1

NOE violations

Number > 0.5 Å none

R.m.s. deviation (Å) 0.065 ± 0.003

Dihedral angle violations

Number > 5° none

R.m.s. deviation (deg) 0.31 ± 0.22

Deviation from idealized covalent geometry

Angles (deg.) 0.51 ± 0.01

Impropers (deg.) 0.370 ± 0.013

Bonds (Å) 0.0035 ± 0.0001

R.m.s. deviation (Å)

Backbone (C', Cα, N) helix segment 4-32 2.10 ± 0.75

all residues 4.68 ± 1.17

All heavy atoms helix segment 4-32 2.85 ± 0.69

all residues 5.75 ± 1.27

Ramachandran dataa (on 569 residues)

Residues in most favoured regions (%) 91.7

Residues in allowed regions (%) 8.0

Residues in generously allowed regions (%) 0.3

Residues in disallowed regions (%) 0.1

aFrom PDB validation server and BMRB Protein Structure Validation Suite.
