# Supplementary material for: Aminoterminal Amphipathic α-Helix AH1 of Hepatitis C Virus Nonstructural Protein 4B Possesses a Dual Role in RNA Replication and Virus Production
Source: PLoS Pathog. 2014 Nov 13;10(11):e1004501. doi: 10.1371/journal.ppat.1004501 (PMC4231108; doi:10.1371/journal.ppat.1004501)
Supplement: Table S2 — Oligonucleotide sequences. (DOCX) [file ppat.1004501.s002.docx]

**Supplementary Table 2. Oligonucleotide sequences.**

| Name | Sequence^1^ |
| --- | --- |
| J6-66-fd | 5’-ACGCAGAAAGCGCCTAGCCAT-3’ |
| HCV-165-rv | 5’-TACTCACCGGTTCCGCAGA-3’ |
| GAPDH-fd | 5’-GAAGGTGAAGGTCGGAGTC-3’ |
| GAPDH-rv | 5’-GAAGATGGTGATGGGATTTC-3’ |
| EMCV-fd | 5’-GGCCTCGGTGCACATGCTTTACA-3’ |
| JFH1-9442-rv | 5'-GTACCTAGTGTGTGCCGCTC-3' |
| JFH1-5042-fd | 5’-GGCCTCACACACATAGACGCC-3’ |
| JFH1-7730-rv | 5’-GTTACTCAAAGGGTTGATTGG-3’ |
| JFH1-6110-fd | 5’-CAATGGATGAACAGGCTTA-3’ |
| JFH4B-220-rv | 5’-AGGGGCGACGTGGTTTCCTCT-3’ |
| AH1-KASK-fd | 5’-TAGCCGAGATGTTGAAG**GC**ATCCAAGATCCAAGGCTTGCT-3’ |
| AH1-KASK-rv | 5’-AAGCCTTGGATCTTGGA**TGC**CTTCAACATCTCGGCTATCC-3’ |
| AH1-KKAA-fd | 5’-GCCGAGATGTTG**GC**GTCC**GC**GATCCAAGGCTTGCTGCAGC-3’ |
| AH1-KKAA-rv | 5’-AAGCCTTGGATC**GC**GGAC**GC**CAACATCTCGGCTATCCGCT-3’ |
| AH1-EEAA-fd | 5’-AGAGGGGCAGCGGATAGCCG**C**GATGTTGAAGTCCAAGAT-3’ |
| AH1-EEAA-rv | 5’-CGGCTATCCGCTGCCCCTCT**G**CGATGAGAGCCGCCCTAG-3’ |
| AH1-K20A-fd | 5’-TGAAGTCC**GC**GATCCAAGGCTTGCTGCAGC-3’ |
| AH1-K20A-rv | 5’-CTTGGATC**GC**GGACTTCAACATCTCGGCTA-3’ |
| AH1-K18A-fd | 5’-AGATGTTG**GC**GTCCAAGATCCAAGGCTTGC-3’ |
| AH1-K18A-rv | 5’-TCTTGGAC**GC**CAACATCTCGGCTATCCGCT-3’ |
| JFHAH2mut-rv1 | 5’-GTGTCTGGCC**GC**A**GC**TTGTTCCACTTTGGGC**GC**TGAAGCCTGCAT-3’ |
| JFHAH2mut-rv2 | 5’-GATGCCGCTAATG**GC**GTTC**GC**CATGTGTCTGGCC**GC**A**GC**-3’ |
| JFHAH2mut-fd1 | 5’-CACATG**GC**GAAC**GC**CATTAGCGGCATCCAA**GC**CCTCGCAGGATTG-3’ |
| JFHAH2mut-fd2 | 5’-AAAGTGGAACAA**GC**T**GC**GGCCAGACACATG**GC**GAAC**GC**C-3’ |
| JFH4BQ26R-rv | 5’-GGGCCTGCTTAGAGGCCTGC**C**GCAGCAAGCCTTGGATCT-3’ |
| JFH4BQ26R-fd | 5’-AGATCCAAGGCTTGCTGC**G**GCAGGCCTCTAAGCAGGCCC-3’ |
| JFH4BQ22R-rv | 5’-TGCTTAGAGGCCTGCTGCAGCAAGCCT**C**GGATCTTGGAC-3’ |
| JFH4BQ22R-fd | 5’-GTCCAAGATCC**G**AGGCTTGCTGCAGCAGGCCTCTAAGCA-3’ |

^1^Mutated nucleotides are shown in bold.
