# Supplementary material for: Aminoterminal Amphipathic α-Helix AH1 of Hepatitis C Virus Nonstructural Protein 4B Possesses a Dual Role in RNA Replication and Virus Production
Source: PLoS Pathog. 2014 Nov 13;10(11):e1004501. doi: 10.1371/journal.ppat.1004501 (PMC4231108; doi:10.1371/journal.ppat.1004501)
Supplement: Figure S1 — Amino acid sequence and superimposition of the backbone heavy atoms (N, Cα, and C′) of the final set of 37 calculated structures for the best overlap of residues (PDB entry 2LVG). This Figure shows that the α-helix is clearly defined between residues Ala 4 and Ala 32 (box), but with an RMSD of 2.1 Å (see Table S1). (DOCX) [file ppat.1004501.s003.docx]

1 5 10 15 20 25 30 35 40

|...|....|....|....|....|....|....|....|

**
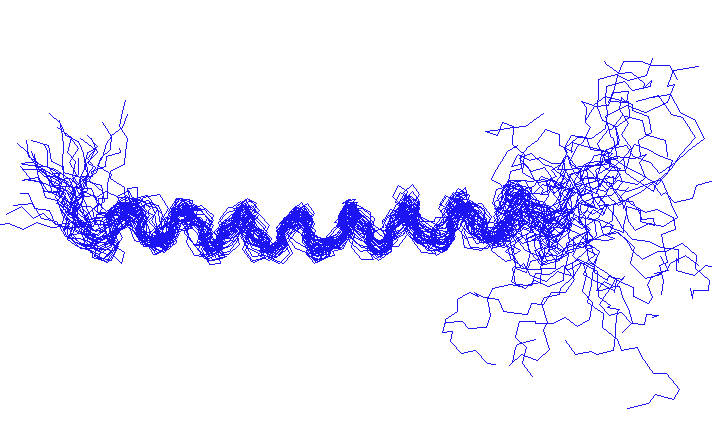
**ASRAALIEEGQRIAEMLKSKIQGLLQQASKQAQDIQPAMQ

*C*

*N*

**Supplementary Figure 1.** Amino acid sequence and superimposition of the backbone heavy atoms (N, Cα, and C') of the final set of 37 calculated structures for the best overlap of residues (PDB entry 2LVG). This Figure shows that the α-helix is clearly defined between residues Ala 4 and Ala 32 (box), but with an RMSD of 2.1 Å (see Supplementary Table 1).
