# Supplementary material for: Aminoterminal Amphipathic α-Helix AH1 of Hepatitis C Virus Nonstructural Protein 4B Possesses a Dual Role in RNA Replication and Virus Production
Source: PLoS Pathog. 2014 Nov 13;10(11):e1004501. doi: 10.1371/journal.ppat.1004501 (PMC4231108; doi:10.1371/journal.ppat.1004501)
Supplement: Figure S2 — H7-T7-IZ cells were transfected with T7 RNA polymerase-driven N3-5B polyprotein expression constructs harboring the indicated mutations in HA-tagged NS4B, followed by selective membrane permeabilization and immunofluorescence microscopy. Cells were subjected to total (0.2% digitonin [Dig 0.2%], upper row) or selective membrane permeabilization (0.05% digitonin [Dig 0.05%], lower row), as described in the Materials and Methods section. Representative pictures for wild-type (wt) and the different mutants are shown. (DOCX) [file ppat.1004501.s004.docx]

*
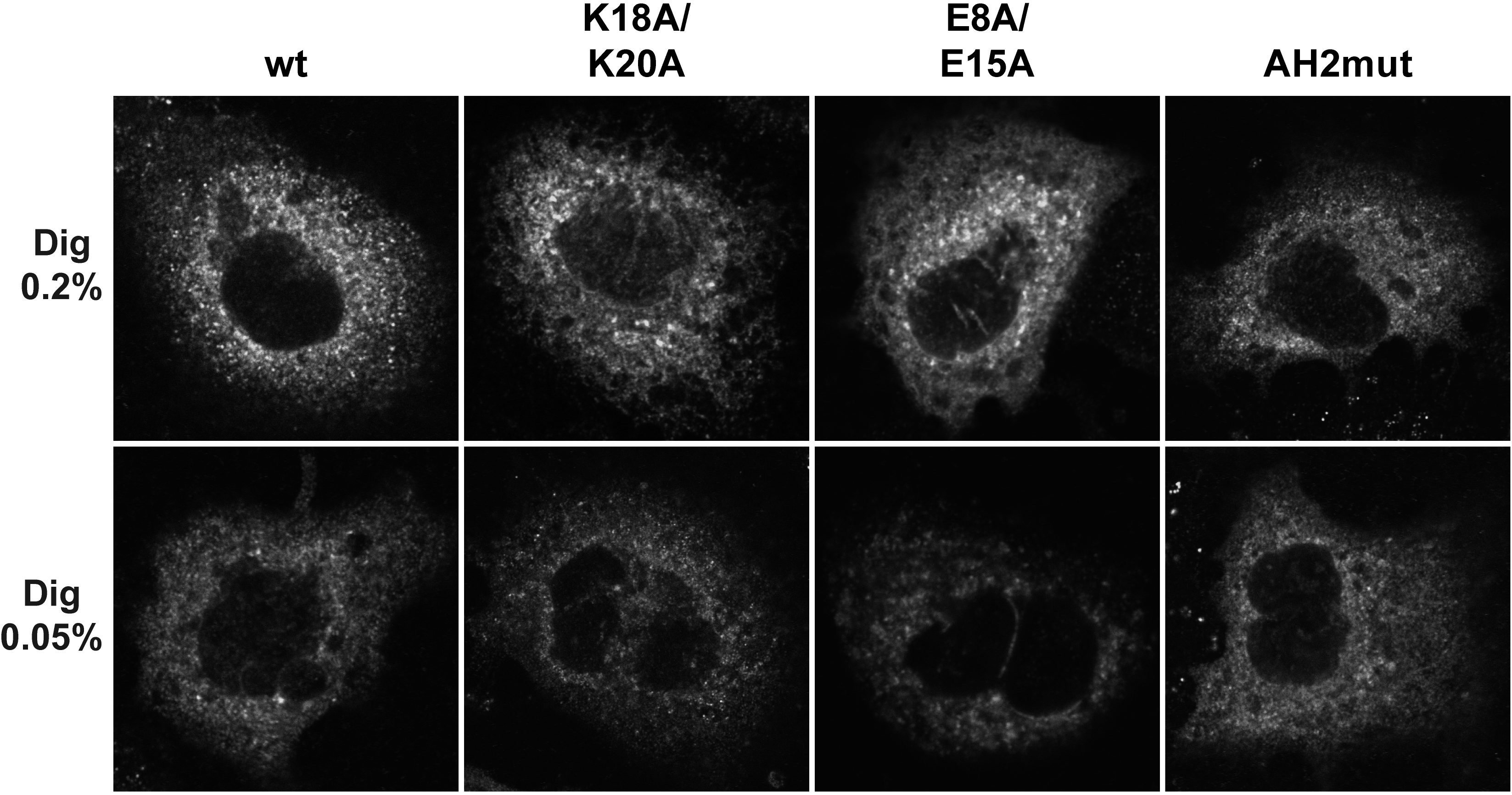
*

**Supplementary Figure 2.** H7-T7-IZ cells were transfected with T7 RNA polymerase-driven N3-5B polyprotein expression constructs harboring the indicated mutations in HA-tagged NS4B, followed by selective membrane permeabilization and immunofluorescence microscopy. Cells were subjected to total (0.2% digitonin [Dig 0.2%], upper row) or selective membrane permeabilization (0.05% digitonin [Dig 0.05%], lower row), as described in the Materials and Methods section. Representative pictures for wild-type (wt) and the different mutants are shown.
